# Supplementary material for: Linkage between endosomal escape of LNP-mRNA and loading into EVs for transport to other cells
Source: Nat Commun. 2019 Sep 24;10:4333. doi: 10.1038/s41467-019-12275-6 (PMC6760118; doi:10.1038/s41467-019-12275-6)
Supplement: Supplementary file 1 — Supplementary Information [file 41467_2019_12275_MOESM1_ESM.pdf]

Linkage between endosomal escape of LNP-mRNA and loading into EVs for transport to other cells

Maugeri et al.

Supplementary information:

Supplementary Table 1. Biophysical characteristics of lipid nanoparticles. Main characteristics of lipid nanoparticles (LNPs) used in this study, which are comprised of ionizable lipid (DLin-MC3-DMA or DLin-DMA), cholesterol, DSPC (1,2-distearoyl-sn-glycero-3-phosphocholine), PEGylated lipid (DMPE-PEG2000) and hEPO-mRNA. Data are shown as mean ± SEM for DD-LNPs (n= 9 independent measurements) and MC3-LNPs (n= 4 independent measurements). PDI: polydispersity index; EE (%): efficiency of encapsulation of the mRNA into LNPs. Size is given as the Z-average diameter. Source data are provided as a Source Data file.

Ionizable lipid	Size (nm)	PDI	mRNA concentration (mg/mL)	Lipid concentration (mg/mL)	EE (%)
DLin-DMA	88 ± 4	0.1 ± 0.01	0.1 ± 0.002	0.97 ± 0.029	94 ± 1.3
DLin-MC3	84 ± 2	0.08 ± 0.02	0.1 ± 0.002	1.02 ± 0.026	97 ± 0.2

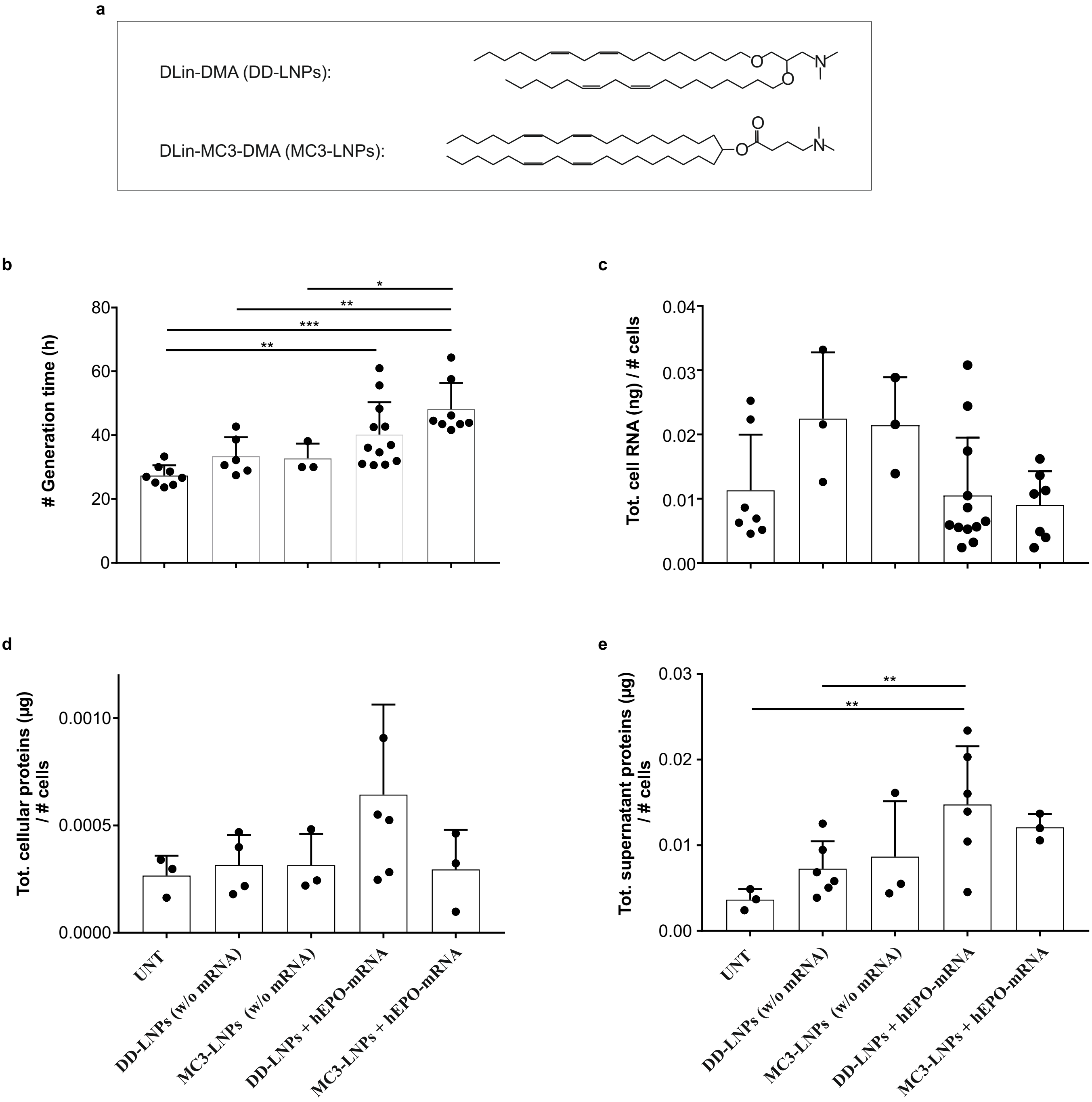

Supplementary Figure 1

Supplementary Fig. 1.

Chemical structure of LNPs and effects of LNPs on cells. DD-LNPs or MC3-LNPs each containing 100µg of hEPO mRNA were transferred to recipient cells in separate experiments. Cells were also treated with empty LNPs (without hEPO mRNA). **(a)** Chemical structures of LNPs with two different ionizable lipids namely DLin-MC3-DMA and DLin-DMA. **(b)** Cell generation time calculated based on the difference between the number of cells at the beginning and at the end of the LNP treatment interval (defined ΔN). Untreated (n=8), DD-LNPs (w/o mRNA) (n=6), MC3-LNPs (w/o mRNA) (n=3), DD-LNPs + mRNA (n=12), MC3-LNPs + mRNA (n=8). (**c)** Total cellular RNA. Untreated (n=7), DD-LNPs (w/o mRNA) (n=3), MC3-LNPs (w/o mRNA) (n=3), DD-LNPs + mRNA (n=12), MC3-LNPs + mRNA (n=7). **(d)** Total cellular proteins. Untreated (n=3), DD-LNPs (w/o mRNA) (n=4), MC3-LNPs (w/o mRNA) (n=3), DD-LNPs + mRNA (n=6), MC3-LNPs + mRNA (n=3). **(e)** Total supernatant proteins. Untreated (n=3), DD-LNPs (w/o mRNA) (n=6), MC3-LNPs (w/o mRNA) (n=3), DD-LNPs + mRNA (n=6), MC3-LNPs + mRNA (n=3). Data are represented as scatter dot plots including mean (bars) and SD of the number (n) of biologically independent samples specified for each panel. Statistically significant difference between each group was assessed by one-way ANOVA followed by Tukey's multiple comparisons test. *$p < 0.05$, **$p < 0.01$ and ***$p < 0.001$. Source data are provided as a Source Data file.

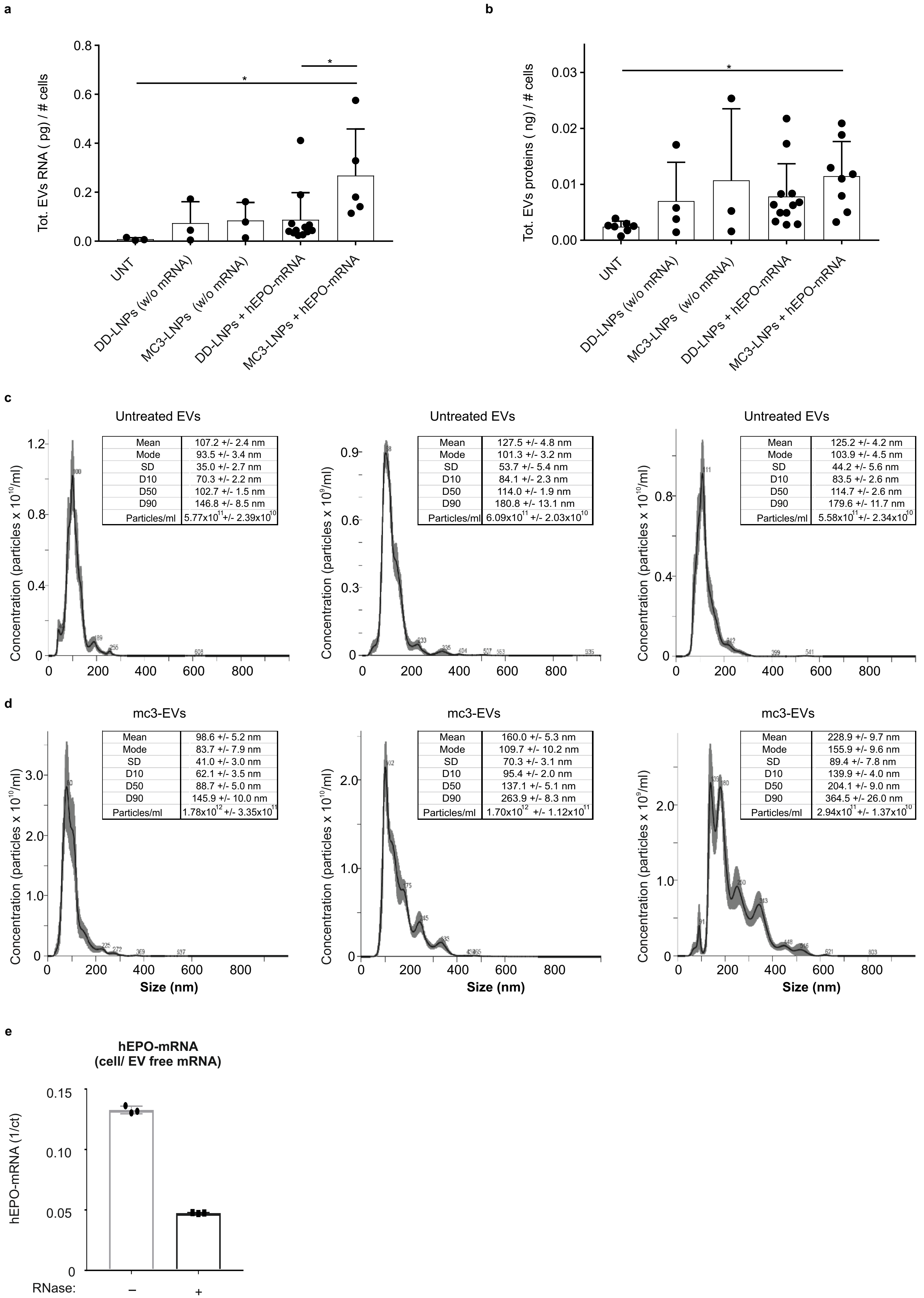

Supplementary Figure 2

Supplementary Fig. 2.

Characterization of EVs. (a) Total EV-RNA normalized to ΔN (difference between the number of cells at the beginning and at the end of the treatment interval). Untreated (n=3), DD-LNPs (w/o mRNA)(n=3), MC3-LNPs (w/o mRNA) (n=3), DD-LNPs + mRNA (n=12), and MC3-LNPs + mRNA (n=5). **(b)** Total EV proteins normalized to ΔN. Untreated (n=7), DD-LNPs (w/o mRNA) (n=4), MC3-LNPs (w/o mRNA) (n=3), DD-LNPs + mRNA (n=12), and MC3-LNPs + mRNA (n=8). Data are represented as scatter dot plots including mean (bars) and SD of the number (n) of biologically independent samples specified for each panel. Statistically significant difference between each group was assessed by using one-way ANOVA followed by Tukey's multiple comparisons test. *p < 0.05, **p < 0.01 and ***p < 0.001.

Upon MC3-LNPs treatment or not, EVs derived from HTB-177 cells were isolated and used for nanoparticle tracking analysis (NTA). **(c)** Size distribution and concentration of EVs from n=3 biologically independent untreated cells **(d)** Size distribution and concentration of EVs from n=3 biologically independent MC3-LNP treated cells. For each graph a table is provided, including Mean and Modal sizes, SD, D10, D50, D90 and particle concentration.

(e) The effect of RNase treatment on control i.e. pure hEPO-mRNA (cell free and EV free). The experiment was performed as n=3 biologically independent samples and the hEPO-mRNA qPCR data is represented as scatter dot plot and mean SD. Source data are provided as a Source Data file.

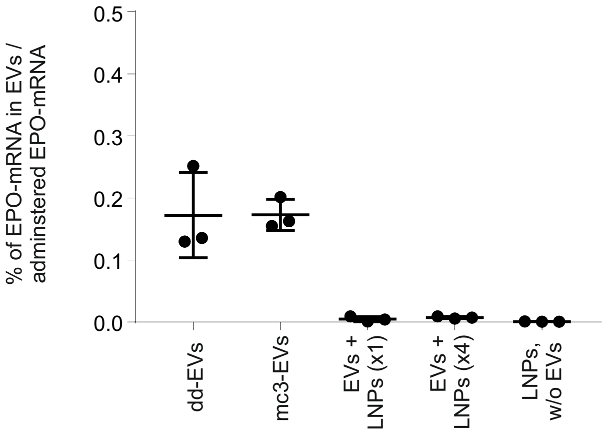

Supplementary Figure 3

Supplementary Fig. 3.

Evaluation of direct transfer of hEPO mRNA from LNPs to EVs. EVs from untreated cells were incubated with MC3-LNPs or DD-LNPs containing hEPO-mRNA (in PBS, *i.e.* in absence of cells). After 2 h of incubation with LNP-hEPO-mRNA, the EVs were re-isolated by ultracentrifugation, total RNA from EVs was isolated and the presence/absence of hEPO mRNA was analyzed by qPCR to evaluate the direct transfer of hEPO mRNA from LNPs into EVs. As a positive control, cells were administered with MC3-LNPs or DD-LNPs containing hEPO-mRNA and EVs were isolated (so called MC3-EVs, or DD-EVs). Results show that hEPO-mRNA was not detected in EVs when directly mixed with LNP-hEPO-mRNA in the absence of cells. By contrast, when same LNP-hEPO-mRNA was administered to cells, the EVs derived from these cells contained hEPO-mRNA. The experiment was performed in biological triplicate. Data are presented as percentage of hEPO mRNA detected in EVs relative to the administered amount of hEPO-mRNA delivered by LNPs to cells or hEPO-mRNA amount directly mixed with EVs. Mean values (bars) with standard deviation (SD) of n=3 biologically independent samples are shown. Source data are provided as a Source Data file.

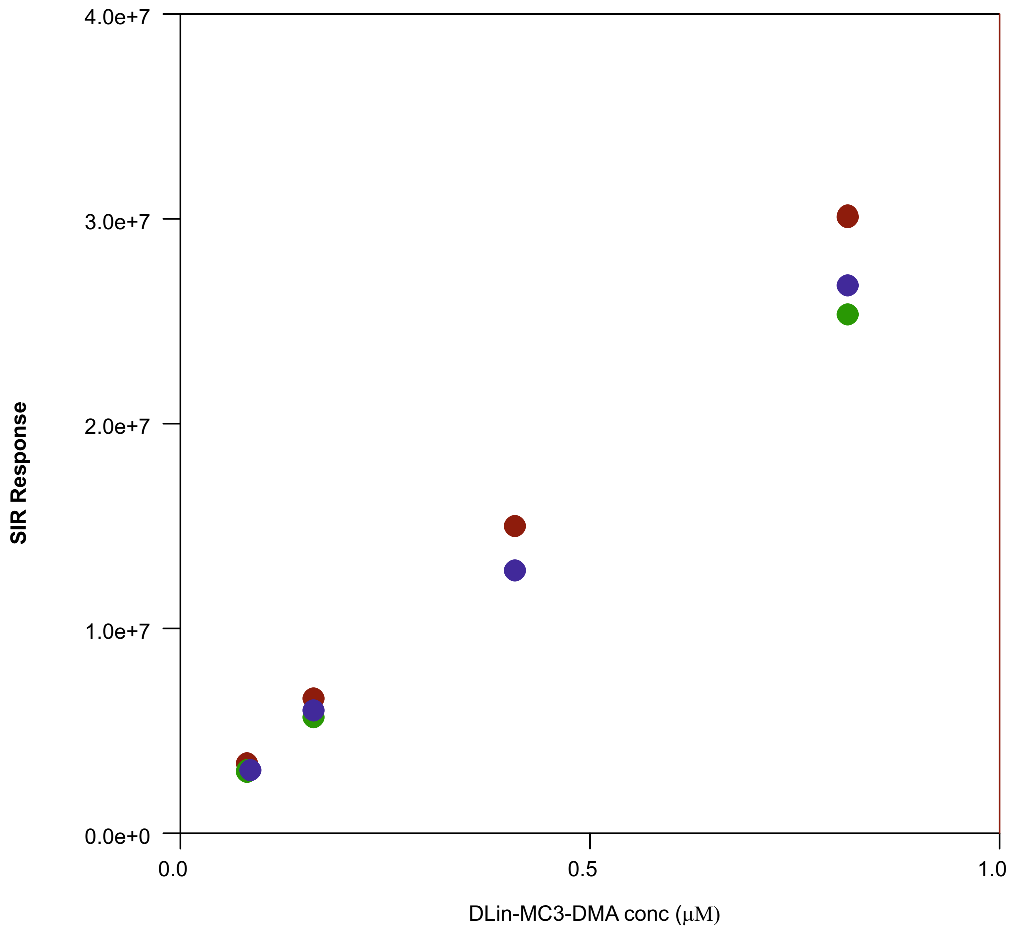

Supplementary Figure 4

Supplementary Fig. 4.

UPLC-MS analysis. DLin-MC3-DMA samples prepared in ethanol (red dots), in 1% (w/w) Triton X-100 (green dots) and in 1% (w/w) Triton X-100 with added fixed amount "empty" EVs (blue dots).

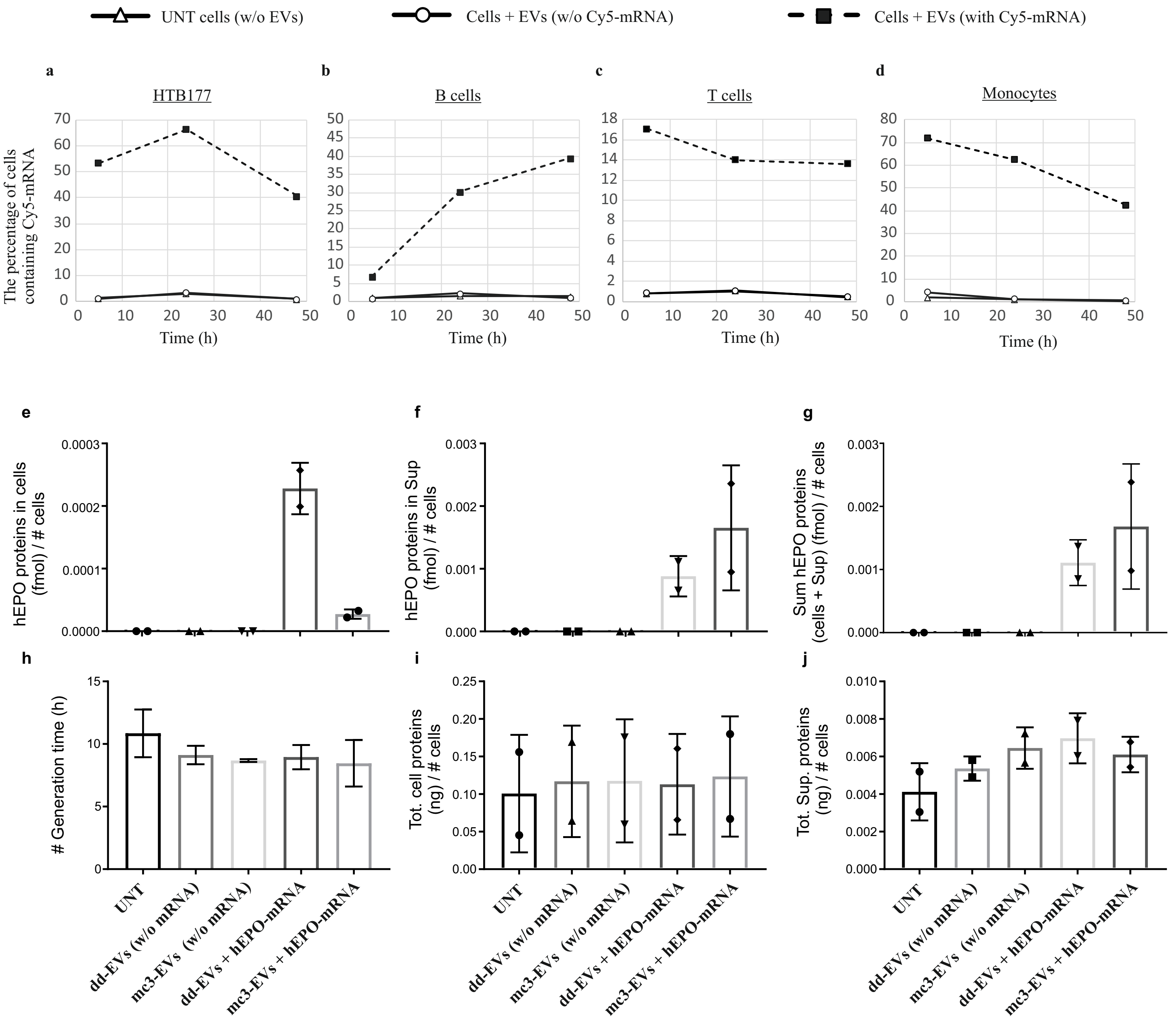

Supplementary Figure 5

Supplementary Fig. 5.

Delivery of mRNA to epithelial and immune cells via EVs. *Delivery of Cy5 mRNA*: DD-LNPs containing Cy5 mRNA were administered to HTB-177 cells. After 96 h of LNP treatment, dd-EVs (isolated from DD-LNP-treated cells) containing Cy5 mRNA were transferred to autologous HTB-177 cells and peripheral blood mononuclear cells (PBMCs). PBMCs were stained with specific surface markers against B-cells (CD19), T-cells (CD3) and monocytes (CD14) after 5, 24 and 48 h of EV delivery, and Cy5 mRNA in recipient cells was detected by FACS. Percentage of cells which uptake (positive for) Cy5-labeled mRNA via EVs, is shown. **(a)** Percentage of HTB-177 cells which uptake Cy5 mRNA, **(b)** Percentage of B-cells, **(c)** Percentage of T-cells and **(d)** Percentage of monocytes positive (uptake) for Cy5 mRNA estimated based on antibody fluorescence and Cy5 fluorescence labelled mRNA after dd-EV transfer to cells at each time point. White triangles-solid line: untreated cells (w/o EVs), white circles-solid line: Cells + EVs (w/o Cy5-mRNA) and black squares-dashed line: Cells + EVs (with Cy5-mRNA).

Delivery of hEPO-mRNA: MC3-EVs incorporated with hEPO-mRNA (from MC3-LNP treated cells), or DD-EVs incorporated with hEPO-mRNA (from DD-LNP treated cells) were transferred to HTB-177 recipient cells. EVs not containing mRNA (obtained from untreated cells) were delivered as control. After 48h, cells and the supernatants were analyzed. Total protein was quantified by Qubit fluorometer and the hEPO protein using ELISA specific for human erythropoietin. **(e, f)** Level of hEPO protein quantified in cells and in supernatants, **(g)** Sum of hEPO protein quantified in cells and supernatant, **(h)** Generation time of recipient cells after a culturing period of 48h with EVs treatment, **(i)** Total cellular proteins and **(j)** Total proteins in supernatants. Data are presented as mean values with standard deviation (SD) of two replicates (n=2) at each time point. Source data are provided as a Source Data file.

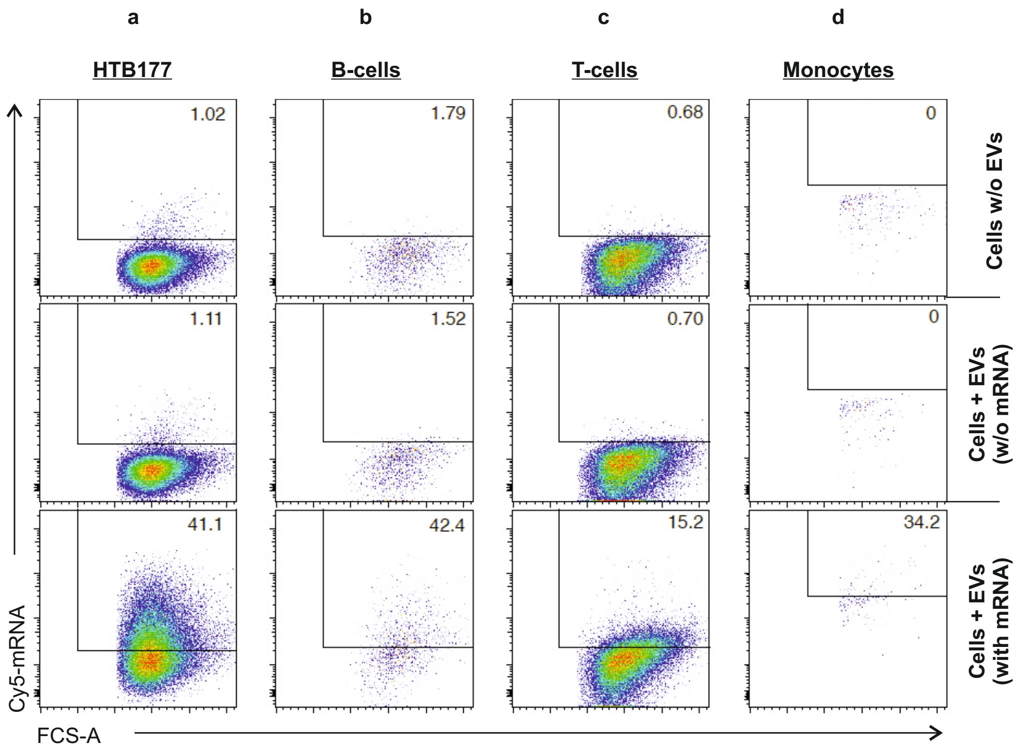

Supplementary Figure 6

Supplementary Fig. 6.

Delivery of Cy5 mRNA to epithelial and primary immune cells via EVs. DD-LNPs containing 76µg of Cy5 mRNA were administrated to HTB-177 cells and EVs from DD-LNP treated cells (DD-EVs) were isolated after 96h of LNPs treatment. 78µg DD-EVs containing Cy5 mRNA were transferred to HTB-177 cells and PBMCs each in independent assays. Cells were harvested after different intervals (2h, 24h, 48h) of EVs transfer and stained with monoclonal antibodies (mAbs) for surface markers against CD19 (B-cells), CD3 (T-cells) and CD14 (monocytes). FACS analysis was performed to detect Cy5 mRNA in recipient cells and each cell type positive for Cy5 mRNA was estimated based on antibody-fluorescence and Cy5-fluorescence. **(a)** Percentage of HTB-177 cells **(b)** B cells, **(c)** T cells and **(d)** monocytes positive for Cy5 mRNA after EVs transfer at 48h. The FACS dot plots represent Cy5 mRNA (y-axis) vs. FCS-A (x-axis) at given time point. Percentage of cells positive for Cy5 mRNA is reported in the upper right quadrant. Cells (w/o EVs): untreated cells. Cells + EVs (w/o mRNA): cells treated with EVs not containing mRNA. Cells + EVs (with mRNA): cells treated with EVs containing the mRNA.

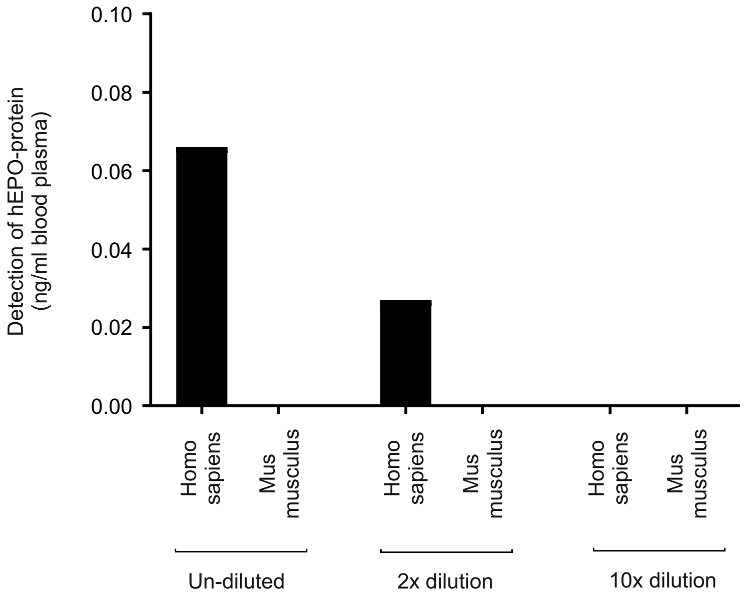

Supplementary Figure 7

Supplementary Fig 7.

Confirmation of specificity of hEPO ELISA kit. The human erythropoietin ELISA Kit was tested to confirm its' specificity for human erythropoietin (hEPO) and to examine its' cross-reactivity with mouse plasma EPO-protein. The plasma from untreated mouse (n=1) and fresh plasma from normal human (n=1) both undiluted and diluted (2x and 10x) were tested. No signals were detected for hEPO in untreated murine plasma. Source data are provided as a Source Data file.

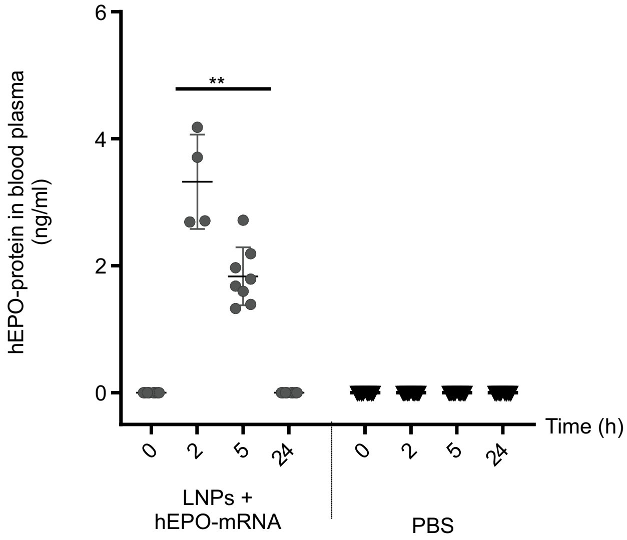

Supplementary Figure 8

Supplementary Fig 8.

Delivery of hEPO mRNA via MC3-LNPs and hEPO protein analysis. Mice were intravenously injected with 100µL of MC3-LNPs, containing 1.5µg of hEPO mRNA (per mouse), or with equivalent volume of PBS as control. The concentrations of hEPO protein in murine plasma were determined by ELISA at 0h, 2h, 5h and 24h after injection. Data are presented as the mean (bars) and standard deviation (SD) from n=8 independent animals, except for the 2h time point (n=4). The plasma hEPO protein from MC3-LNP delivery was compared between 2h and 5h by unpaired two-tailed Student's *t*-test with significant P-value **p < 0.01. Source data are provided as a Source Data file.

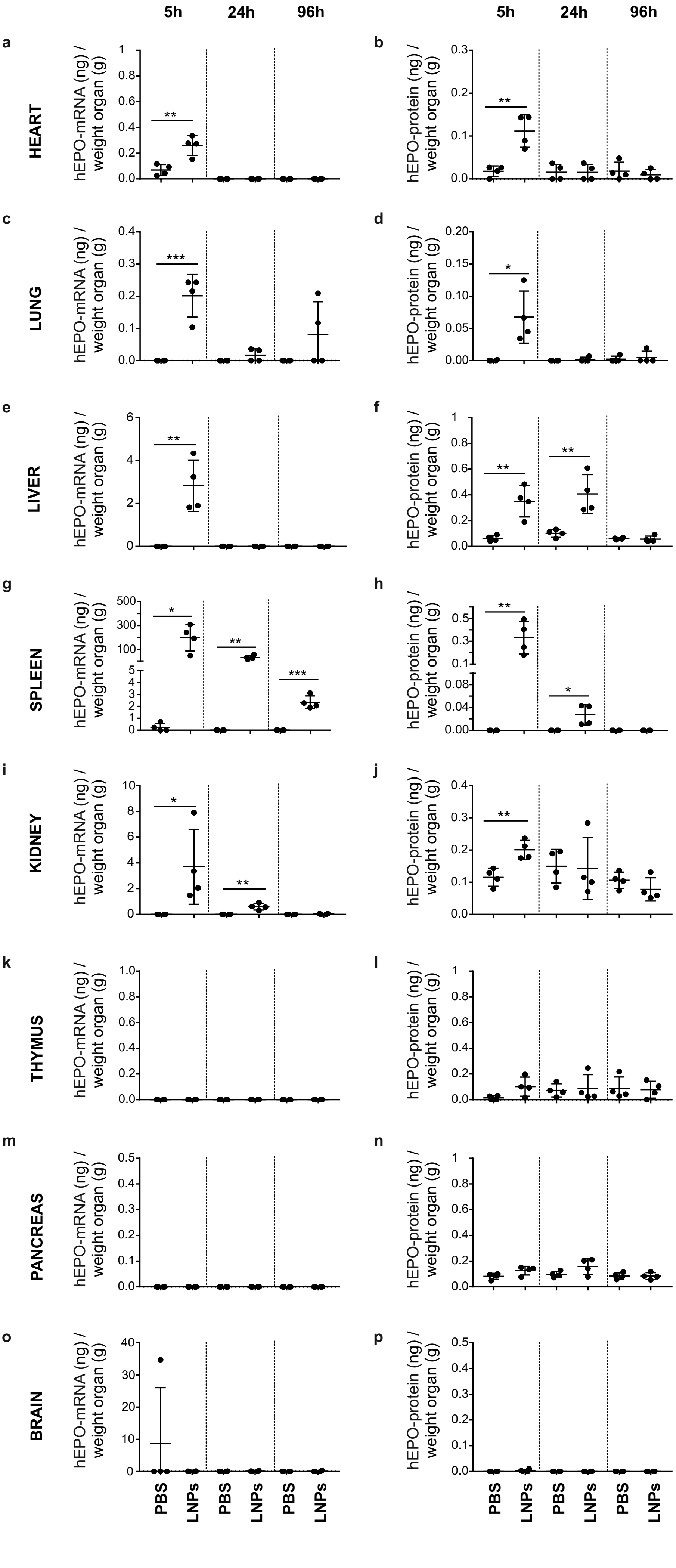

Supplementary Figure 9

Supplementary Fig. 9.

HEPO mRNA and protein quantification in murine organs after LNP delivery. Mice were intravenously injected with 100µl of MC3-LNPs, containing 1.5µg of hEPO mRNA (per mouse), or with equivalent volume of PBS as control. The levels of hEPO mRNA and hEPO protein in eight different organs after 5h, 24h and 96h of injection were determined by qPCR and ELISA respectively. **(a, b)** Levels of hEPO mRNA and protein in heart, **(c, d)** lung, **(e, f)** liver, **(g, h)** spleen, **(i, j)** kidney, **(k, l)** thymus, **(m, n)** pancreas and **(o, p)** brain. Data is represented as total amount of hEPO mRNA or protein detected in entire organ normalized to organ weight. Data are presented as mean values (bars) with standard deviation (SD) of n=4 independent animals, at each time point. For each time point in each organ, the MC3-EVs and PBS groups were compared using unpaired two-tailed Student's *t*-test. *p < 0.05, **p < 0.01 and ***p < 0.001. Source data are provided as a Source Data file.

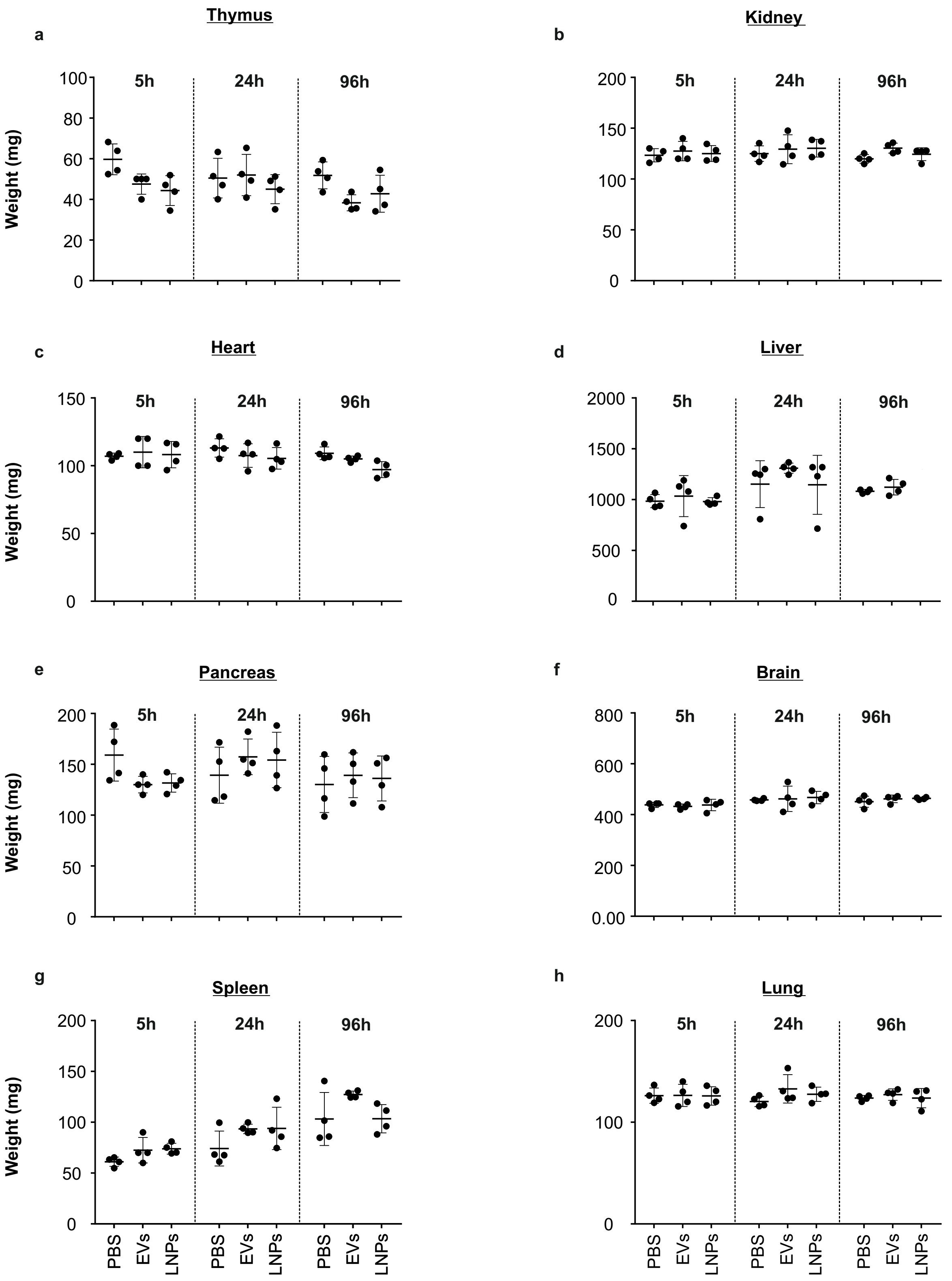

a
Thymus
b
Kidney
c
Heart
d
Liver
e
Pancreas
f
Brain
g
Spleen
h
Lung
Weight (mg)
5h
24h
96h
PBS
EVs
LNPs
0
20
40
60
80
100
50
150
200
500
1000
1500
2000
0.00
400
600
800

Supplementary Fig. 10.

Comparison of organs' weight. The weight of each mouse determined after 5h, 24h and 96h of MC3-EVs or MC3-LNPs injection. No considerable differences were observed. **(a)** Thymus, **(b)** kidney, **(c)** heart, **(d)** liver, **(e)** pancreas, **(f)** brain, **(g)** spleen and **(h)** lung. Data is shown as mean values (bars) with standard deviation (SD) of n=4 independent animals. Statistical analysis was performed using one-way ANOVA followed by Tukey's multiple comparisons test. Source data are provided as a Source Data file.

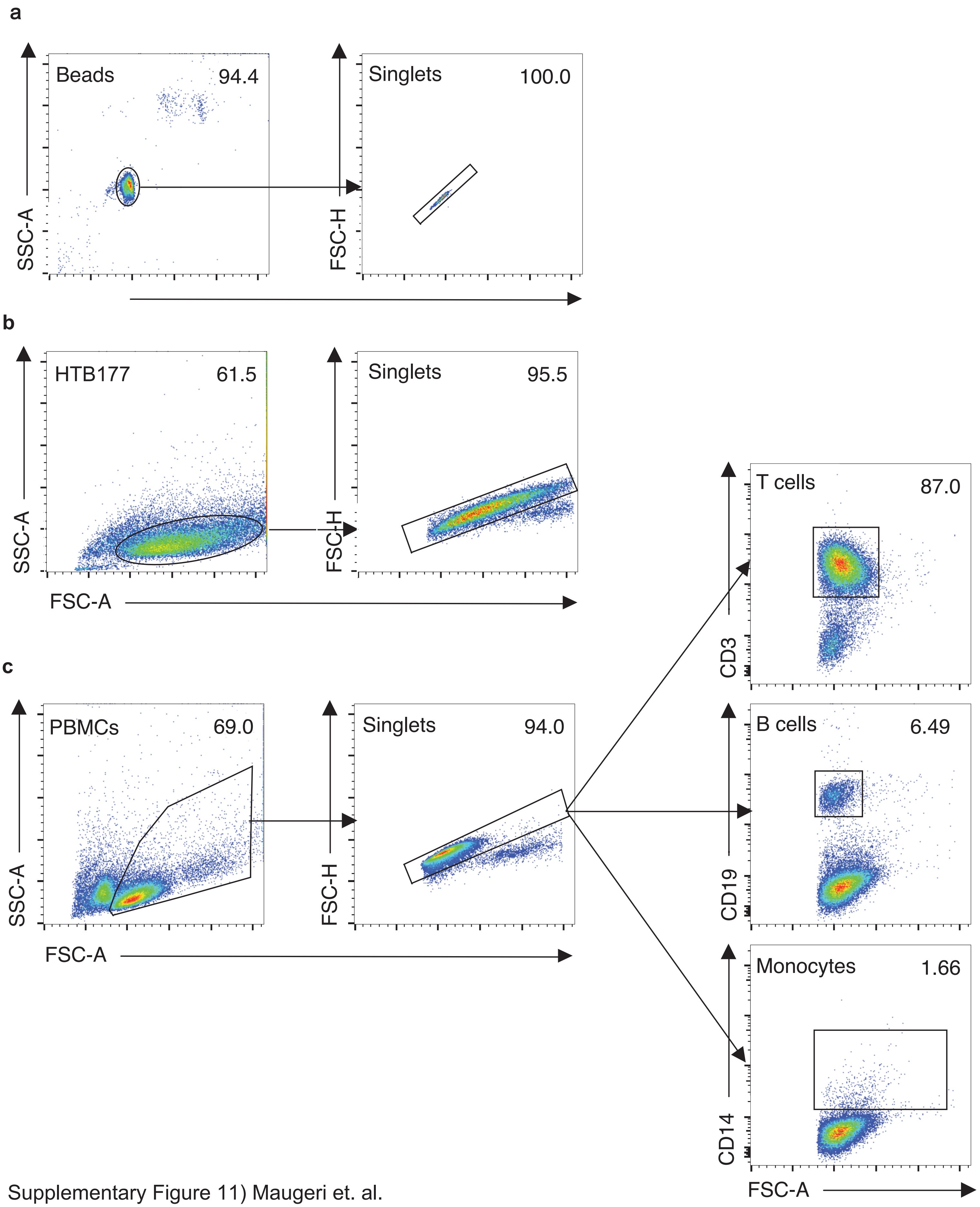

Supplementary Figure 11) Maugeri et. al.

Supplementary Fig. 11.

Gating strategy for beads and cells. (a) The beads were gated using FSC-A and SSC-A and doublets were excluded. **(b)** HTB-177 and **(c)** PBMCs were gated using FSC-A and SSC-A and doublets were excluded. PBMCs were further gated on T cells ($CD3^+$), B cells ($CD19^+$) and monocytes ($CD14^+$).
